# Supplementary material for: Enhanced Photocatalytic Hydrogen Production of ZnIn2S4 by Using Surface-Engineered Ti3C2Tx MXene as a Cocatalyst
Source: Materials (Basel). 2023 Mar 8;16(6):2168. doi: 10.3390/ma16062168 (PMC10059248; doi:10.3390/ma16062168)
Supplement: Supplementary file 1 [file materials-16-02168-s001.zip › materials-2256071-supplementary.pdf]

Supplementary Materials

Enhanced Photocatalytic Hydrogen Production of $ZnIn_2S_4$ by Using Surface-Engineered $Ti_3C_2T_x$ MXene as a Cocatalyst

Mengdie Cai [*,†], Xiaoqing Zha [†], Zhenzhen Zhuo, Jiaqi Bai, Qin Wang, Qin Cheng, Yuxue Wei and Song Sun [*]

School of Chemistry and Chemical Engineering, Anhui University, Hefei 230601, China
* Correspondence: caimengdie1987@163.com (M.C.); suns@ustc.edu.cn (S.S.)
† These authors contributed equally to this work.

1. Experimental

Theoretical Calculation

Cambridge Sequential Total Energy Package (CASTEP) was used to perform all the density functional theory (DFT) calculations within the spin-polarized generalized gradient approximation (GGA) with Perdew-Burke-Ernzerhof (PBE) exchange-correlation functional. The plane-wave energy cutoff was set to 400 eV, and gamma point grid was employed for the Brillouin zone sampling. The convergence criterion of energy and force calculations were set to 10-5 eV/atom and 0.03 eV $Å^{-1}$, respectively. The surface computations were performed by using the slab model. The $Ti_3C_2F_2$ (001), $Ti_3C_2(OH)_2$ (001), and $Ti_3C_2O_2$ (001) slabs were built to calculate their work functions.

2. Results

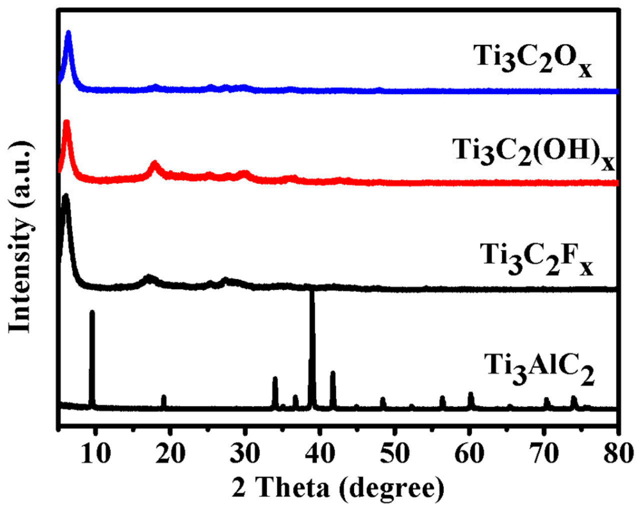

Figure S1. XRD patterns of the Ti_3AlC_2 and as-synthesized $Ti_3C_2F_x$, $Ti_3C_2(OH)_x$ and $Ti_3C_2O_x$.

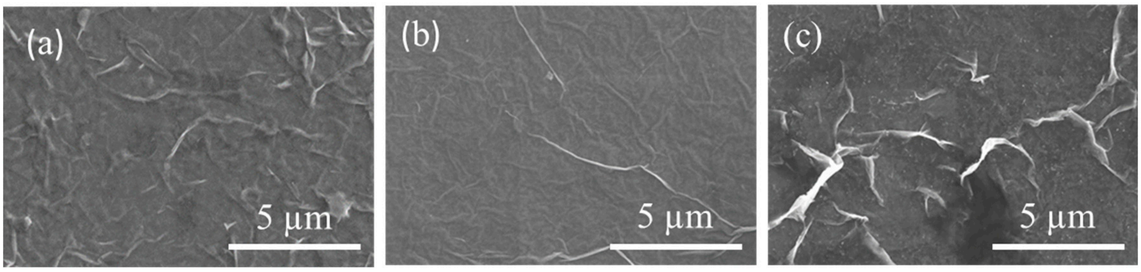

Figure S2. (a) SEM of $Ti_3C_2F_x$, (b) $Ti_3C_2(OH)_x$, and (c) $Ti_3C_2O_x$.

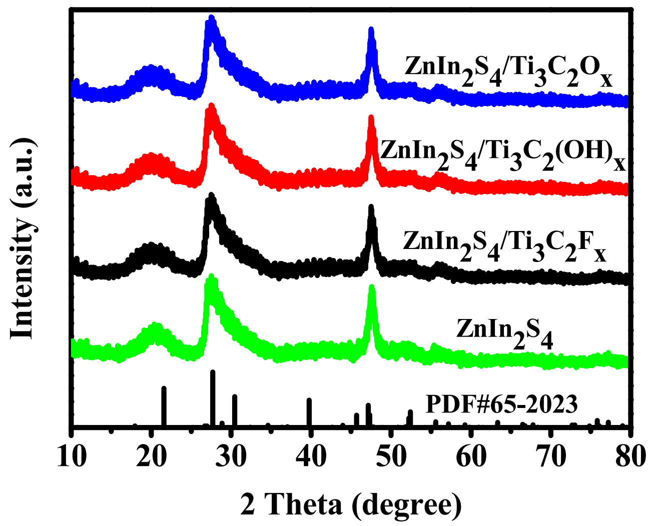

Figure S3. XRD pattern of $ZnIn_2S_4$ and $ZnIn_2S_4/Ti_3C_2T_x$ (T=F, OH and O).

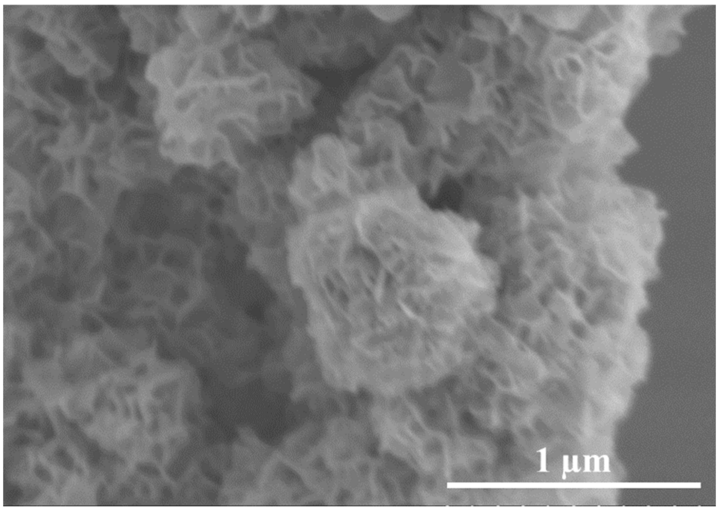

Figure S4. SEM image of $ZnIn_2S_4$

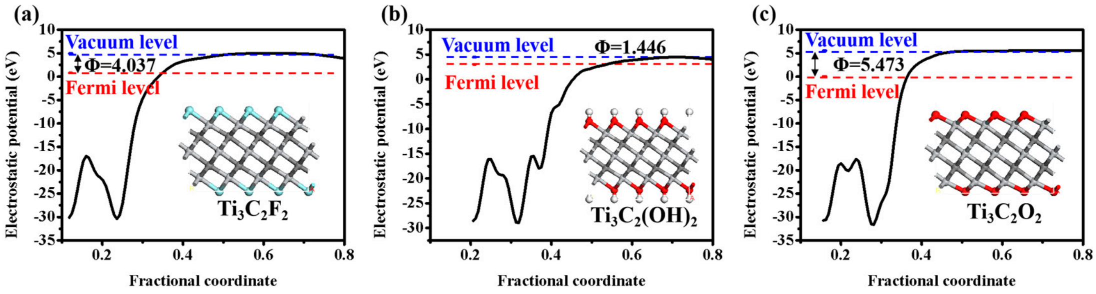

Figure S5. Electrostatic potentials of $Ti_3C_2F_x$, $Ti_3C_2(OH)_x$ and $Ti_3C_2O_x$.

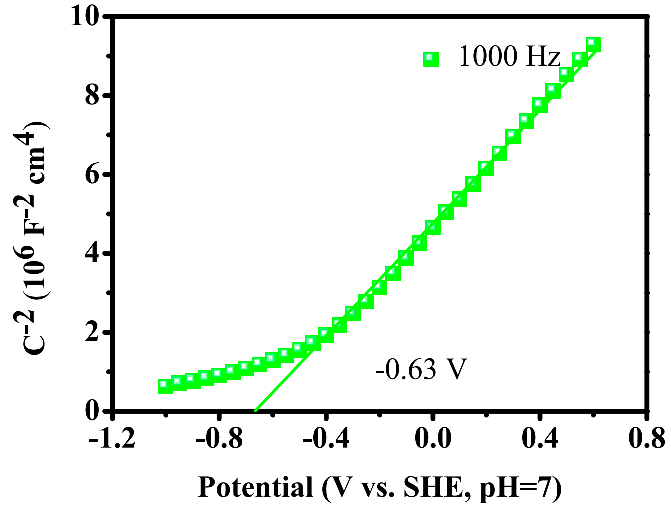

Figure S6. Mott-Schottky diagram of $ZnIn_2S_4$.

To further clarify the photocatalytic mechanism for the $ZnIn_2S_4/Ti_3C_2O_x$, the conduction band (CB) potential (ECB) of the parent $ZnIn_2S_4$ was estimated by the Mott-Schottky method. As shown in Figure S5, the typical Mott-Schottky plot of $ZnIn_2S_4$ revealed an n-type semiconductor feature [1,2]. The flat-band potential of ZnIn2S4 was calculated to be -0.02 V versus Ag/AgCl (i.e. -0.39 V vs. NHE) and the conduction band potential (ECB) can be calculated to be -0.12 V, the corresponding valence band (VB) potential (EVB) can be calculated to be +2.52 V.

Table S1. The atomic ratio of $Ti_3C_2T_x$ (T=F, OH and O) by XPS results

Sample	F(%)	O(%)	Ti(%)	C(%)
$Ti_3C_2F_x$	14.73	23.78	29.45	32.04
$Ti_3C_2(OH)_x$	7.07	30.78	28.86	32.58
$Ti_3C_2O_x$	7.5	31.74	27.36	33.40

Reference

1. Xie, Y.; Liu, Y.; Cui, H.; Zhao, W.; Yang, C.; Huang, F. Facile solution-based fabrication of $ZnIn_2S_4$ nanocrystalline thin films and their photoelectrochemical properties. *Journal of Power Sources* **2014**, 265, 62-66.
2. Liu, Q.; Lu, H.; Shi, Z.; Wu, F.; Guo, J.; Deng, K.; Li, L. 2D $ZnIn_2S_4$ nanosheet/1D TiO_2 nanorod heterostructure arrays for improved photoelectrochemical water splitting. *ACS applied materials & interfaces* **2014,** 6, 17200-17207.
